# Metabarcoding Analysis of Fungal Diversity in the Phyllosphere and Carposphere of Olive (*Olea europaea*)

**DOI:** 10.1371/journal.pone.0131069

**Published:** 2015-07-01

**Authors:** Ahmed Abdelfattah, Maria Giulia Li Destri Nicosia, Santa Olga Cacciola, Samir Droby, Leonardo Schena

**Affiliations:** 1 Dipartimento di Agraria, Università Mediterranea di Reggio Calabria, Località Feo di Vito, 89124, Reggio Calabria, Italy; 2 Dipartimento di Gestione dei Sistemi Agroalimentari e Ambientali, Università degli Studi, Via S. Sofia 100, 95123, Catania, Italy; 3 Department of Postharvest Science ARO, The Volcani Center, Bet Dagan, 50250, Israel; Korea University, REPUBLIC OF KOREA

## Abstract

The fungal diversity associated with leaves, flowers and fruits of olive (*Olea europaea*) was investigated in different phenological stages (May, June, October and December) using an implemented metabarcoding approach. It consisted of the 454 pyrosequencing of the fungal ITS2 region and the subsequent phylogenetic analysis of relevant genera along with validated reference sequences. Most sequences were identified up to the species level or were associated with a restricted number of related *taxa* enabling supported speculations regarding their biological role. Analyses revealed a rich fungal community with 195 different OTUs. *Ascomycota* was the dominating phyla representing 93.6% of the total number of detected sequences followed by unidentified fungi (3.6%) and *Basidiomycota* (2.8%). A higher level of diversity was revealed for leaves compared to flowers and fruits. Among plant pathogens the genus *Colletotrichum* represented by three species (*C*. *godetiae syn*. *C*. *clavatum*, *C*. *acutatum s*.*s* and *C*. *karstii*) was the most abundant on ripe fruits but it was also detected in other organs. *Pseudocercospora cladosporioides* was detected with a high frequency in all leaf samples and to a less extent in ripe fruits. A much lower relative frequency was revealed for *Spilocaea oleagina* and for other putative pathogens including *Fusarium* spp., *Neofusicoccum* spp., and *Alternaria *spp. Among non-pathogen *taxa*, *Aureobasidium pullulans*, the species complex of *Cladosporium cladosporioides* and *Devriesia* spp. were the most represented. This study highlights the existence of a complex fungal consortium including both phytopathogenic and potentially antagonistic microorganisms that can have a significant impact on olive productions.

## Introduction

Among the different fruit tree species, olive (*Olea europaea*) is one of the most important crops on a global scale and its cultivation is rapidly extending due to the growing awareness of the health benefits associated with olive oil consumption. Currently, the available data on fungal communities inhabiting olive phyllosphere and carposphere is very limited and mostly related to plant pathogens, while almost nothing is known about other fungi [1]. Major fungal pathogens attacking olive canopy are *Spilocaea oleagina*, *Colletotrichum* spp., and *Pseudocercospora cladosporioides*, responsible for peacock spot, anthracnose and cercosporiosis, respectively [2]. Other fungal pathogens belonging to the family *Botryosphaeriaceae* and to the genus *Fusarium* can occasionally cause rots on olive drupes [3]. Apart from plant pathogens, several other fungal species have been associated with the olive sooty mold—i.e. a consequence of the epiphytic growth of different saprophytic fungi forming a black film covering leaves, fruits, twigs and branches [3].

Insights into the ecology, identification and quantification of olive pathogens as well as other epiphytic microflora will facilitate the understanding of the complex interactions between the plant and the resident fungal microflora, including potential pathogens and their antagonists. This information may have important practical implications on the management of phyllosphere and carposphere “microenvironments” since many microorganisms exert a competitive action against pathogens and/or induce mechanisms of resistance in the host. Therefore, accurate knowledge of the composition of fungal microbiota may be essential to develop effective biological disease control strategies that are environment-friendly and safe for consumers. These aspects are becoming more and more important in the public opinion and government policies as evidenced by the growing diffusion of organic agriculture.

The massive sequencing of polymerase chain reaction (PCR) amplicons from specific barcode genes represents a powerful culture-independent technique to investigate the whole microbial diversity and determine the relative quantity of community members in environmental samples [4]. This technique may be properly referred to as amplicon metagenomics or metabarcoding and, in the case of fungal communities, is almost exclusively based on the amplification of the ITS regions of the ribosomal DNA (rDNA) [5–7]. A major drawback of the ITS regions concerns difficulties in discriminating phylogenetically related species that may have sequences which are identical or differ only by a few nucleotide positions. This weakness is emphasized in recent high-throughput sequencing approaches since they are commonly based either on the ITS1 or ITS2 regions due to restrictions in sequence read lengths [8]. Consequently, utilized bioinformatics tools and genetic databases enable a good identification of microorganisms up to the level of the genus but they become less reliable when used to identify fungal species. This is particularly true for members of the phylum *Ascomycota* and becomes particularly relevant for fungal plant pathogens since related species with very similar ITS sequences can be characterized by completely different pathogenetic behavior.

Despite the above mentioned drawbacks, the use of the ITS as barcode gene in fungal metabarcoding represents, at least for the moment, a forced choice since this gene can be easily amplified and sequenced with universal primers and largely prevails in GenBank and other databases [9–11]. The choice of either ITS1 or ITS2 is optional since these regions share many properties, and enable similar levels of discrimination [12]. However, ITS2 is generally easier to align because it is less variable in length and lacks the problem of co-amplification of a 5’ SSU intron that is common in many *Ascomycota* [4]. Furthermore, the ITS2 is better represented than ITS1 in databases [13].

In recent years, metagenomic analyses have been increasingly utilized to investigate microbial diversity in a number of different environments and, in most of the cases, they have revealed a vast previously unknown microbial biodiversity missed by conventional cultivation-based methods. A large part of these studies have focused on soils and rhizosphere environments [14–16]. On the other hand, the plant canopy environment has not been widely investigated and only few studies have focused on phyllosphere endophytes [17] while, to the best of our knowledge, fungal biodiversity has been recently investigated only in tomato, grape leaves and balsam poplar [18, 19].

The aim of the present study was the use of the ITS2 region in a metabarcoding approach to investigate the fungal microbiota of the olive phyllosphere and carposphere in different phenological phases. Representative ITS2 sequences were phylogenetically analyzed along with selected reference sequences to enable the most accurate possible identification of putative species.

## Materials and Methods

### Ethics Statement

No specific permits were required for the described field studies. This study did not involve endangered or protected species.

### Sampling and DNA Extractions

Samples of flowers, leaves, fertilized fruitlets and asymptomatic and symptomatic (rotted) fruits were collected in four phenological stages (May 28; June 29; October 17; and December 12) from nine different trees located in three different farms of the Gioia Tauro Plain (Calabria, Italy) with GPS coordinates 38°22'53.0"N and 15°56'27.5"E, 38°22'15.1"N and 15°55'38.3"E and 38°24'44.6"N and 15°56'23.1"E, respectively (Table 1). Investigated farms shared the same olive cultivar (Ottobratica) and were representative of the Gioia Tauro plain, an olive-growing area with over 32,000 Ha, where epidemic outbreaks of anthracnose caused by *Colletotrichum* spp. occur on a yearly basis [20]. Symptoms on symptomatic fruits collected in December were considered to be specific of *Colletotrichum* infections, although the role of other fungal pathogens in fruit rots was not completely excluded.

**Table 1:** Summary of analyses and results of field surveys conducted with different olive tissues collected in four phenological phases from nine different trees located in three different farms.

Sampling date	Sample type	MIDs	Sequences	SRA*	OTUs(Total)**	OTUs (2800)***	Chao1 estimate	Shannon diversity
28.05.12	Leaves	MID3	3335	SRX821225	71	66	93.95	3.78
	Flowers	MID4	9551	SRX1025531	67	42	64.04	2.76
29.06.12	Leaves	MID5	4083	SRX1025537	72	66	95.70	3.63
	Fertilized fruits	MID2	2801	SRX1025554	44	44	91.50	2.163
17.10.12	Leaves	MID10	4816	SRX1025575	85	70	101.24	3.68
12.12.12	Leaves	MID16	12301	SRX1025576	117	88	109.52	3.61
	Asymptomatic fruits	MID19	14610	SRX1025577	88	49	78.22	1.76
	Symptomatic fruits	MID28	6748	SRX1025578	37	30	35.85	1.08

*Sequence Read Archive (SRA) accession numbers for sequences deposited in the BioProject database (NCBI) as PRJNA270912 (http://www.ncbi.nlm.nih.gov/bioproject/270912)

**Total number of detected OTUs

***Number of OTUS detected with an even sequencing depth of 2800 sequences.

A total of 72 samples (3 fields x 3 trees x 8 sample types) were collected during 2013 (Table 1). Each sample consisted of 30–200 g of tissues according to the type of plant part and was harvested around the entire canopy at a height of approximately 2 m in order to cover all cardinal directions. Samples were collected in sterile plastic bags and maintained at 4°C for 4–5 hours before lyophilization. To facilitate the lyophilization, leaves were cut into small pieces of approximately 5–10 mm. Similarly, the flesh of drupes collected in December was cut and separated from the stone. Fertilized fruitlets were lyophilized without any additional treatment. Total DNA was extracted from lyophilized samples as described by Mosca and co-workers [21] and purified using chromatography columns according to Ruano-Rosa and co-workers [22]. The quantity and quality of purified DNA extracts were determined using a Nanodrop 2000 spectrophotometer (Nano-drop Technologies, Wilmington, DE) and by electrophoresis on a 1.2 agarose gel.

### Fungal DNA Amplification

DNA extracts obtained from the collected samples (72) were amplified in triplicate, using the universal fungal primers ITS3-ITS4 to amplify the ITS2 region of the ribosomal DNA [23]. Both primers were modified to construct fusion primers appropriate for 454 sequencing with adapter sequences A and B, a key sequence and multiplex identifiers (MIDs) (http://www.454.com/). Primers labelled with different MIDs were utilized during amplifications to identify the eight sample types collected (Table 1). In all amplification set specific negative control reactions with water replacing template DNA were used.

PCR reactions were conducted in a total volume of 25 µl containing 2.5μl 10X of reaction buffer, 0.25 μl of each primer (10μM), 0.1μl of DNA of AccuPrime Taq DNA Polymerase High Fidelity (Invitrogen, CA, USA) and 1μl of DNA template (100 ng/µl). Reactions were incubated in an Eppendorf Mastercycler gradient (Hamburg, Germany) for 1 min at 94°C followed by 30 cycles of 30s at 94°C, 30 s at 55°C and 30 s at 68°C. All reactions ended with a final extension of 1 min at 68°C. Triplicate PCR products from each sample were combined and purified with agarose Gel Extract Mini (5 Prime-USA). After purification, concentration and quality were evaluated spectrophotometrically and by gel electrophoresis. Ten µl of each purified sample were pooled together and sequenced by Macrogen Inc. (Seoul, Korea) using 454 GS FLX+ System (Roche Diagnostics Corporation).

Data were deposited in the BioProject database (NCBI) as PRJNA270912 (http://www.ncbi.nlm.nih.gov/bioproject/270912) (Table 1).

### Data Analysis and Statistics

Data analysis was conducted using the bioinformatics pipeline QIIME v. 1.8 [24]. De-multiplexing and quality filtering analyses were done using a minimum quality score of 25, a minimum/maximum length ratio of 200/1000 and a maximum number of homopolymer bases of 6. Additionally, the sliding window test of quality scores (-w) was enabled with a value of 50 to discard sequences with bad windows according to the "-g" command. Sequences were denoised using the denois wrapper [25] and the ITS2 region was extracted using ITSx software [26]. Chimeric sequences were identified and filtered using USEARCh 6.1 [27]. The most abundant sequences were picked as representative sequences to be used in Operational taxonomical units (OTUs) picking and taxonomy assignments. OTUs were picked using the BLAST method [28] and the UNITE dynamic database released on February 2, 2014 (http://unite.ut.ee/). The same database was also utilized for taxonomy assignments [28] using a sequence similarity threshold of 0.97 and maximum e-values of 0.001 and 1e-10 in picking OTUs and in taxonomy assignments, respectively. The taxonomic assignments and the operational taxonomical unite map (OTU map) were used to create the OTU table needed to construct the heat-map and the taxa summaries.

Since the rarefaction plots of the entire OTU table as a function of the sequencing effort with a maximum of 6000 sequences per sample revealed heterogeneity in sampling, the OTU table was rarefied to even sequencing depth of 2800 sequences to remove sample heterogeneity. Weighted and unweighted UniFrac metrics were utilized to evaluate Beta diversity [29]. Alfa diversity was determined by Shannon’s Diversity Index and Chao1 estimate. Beta diversity served to construct UPGMA trees and PCoA plots. The uncertainty in the UPGMA tree was estimated by performing jack-knifing at a depth of 2000 sequences. Trees were visualized and edited in Mega6 [30].

To highlight shared phylotypes, Venn Diagrams were created using the OTUs table created in QIIME and visualized on the website http://bioinfogp.cnb.csic.es/tools/venny/index.html [31].

### Identification of Fungal Species

In order to confirm the accuracy of taxonomic assignments, sequences associated with each OTU within each identified fungal genus, were extracted and introduced in ElimDupes (http://hcv.lanl.gov/content/sequence/ELIMDUPES/elimdupes.html) to detect multiple identical sequences and determine their frequency. Unique representative sequences defined as sequence types (STs), i.e. distinct and reproducible ITS2 sequences recovered in this study, were than manually blasted to identify the closest available reference sequences in the complete NCBI nucleotide collection (http://blast.ncbi.nlm.nih.gov/Blast). Furthermore, ITS2 sequences of the most abundant fungal genera according to the QIIME taxonomic assignments (*Aureobasidium* spp., *Colletotrichum* spp., *Cladosporium* spp., *Pseudocercospora* spp., and *Devriesia* spp.) were phylogenetically analyzed. STs were analyzed along with genetically closely related reference sequences of the same genus to determine their phylogenetic collocation and enable their identification with the highest possible level of accuracy. Before analysis, validated panels of reference ITS2 sequences of *Colletotrichum* acutatum s.l. [32–34], *C*. *boninense s*.*l*. [35], *Pseudocercospora* spp. [36], *Devriesia* spp. [37], *Cladosporium* spp. [38] and *Aureobasidium* spp. [39] were analyzed with the software ElimDupes to delete multiple identical sequences. Some identical reference sequences were included in the panel because they were representative of different species. When none of the above-validated reference sequences was identical to sequences identified in the present study, eventual more closely related sequences were identified by BLAST analyses. Despite being low abundant, a similar analysis was also performed for the genus *Spilocaea* in light of its relevance as olive fungal pathogen [2]. In this case, reference sequences were downloaded from GenBank because of the lack of a validated panel of reference sequences.

For each genus, STs identified in the present study and reference sequences were aligned using MUSCLE and introduced to MEGA for phylogenetic analysis with the Maximum Likelihood method using the Tamura-Nei model [30]. Analyses were performed with 1000 bootstrap replications.

## Results

### Fungal Diversity and Richness

A fragment of the expected size (≅340 bp) was obtained from all olive samples investigated in the present study. The complete panel of amplicons obtained from 8 different olive sample types yielded a total of 151,671 reads with an average length of 337bp. After quality evaluations (length trimming, denoising, ITS2 extraction and chimeric sequence exclusion) a total of 58,245 high quality sequences were recovered and assigned to 195 OTUs. The number of detected sequences ranged from 14,610 in asymptomatic fruits collected in December (DAFr) and 2,801 in fertilized fruitlets collected in June (JFr) (Table 1).

**Fig 1:**
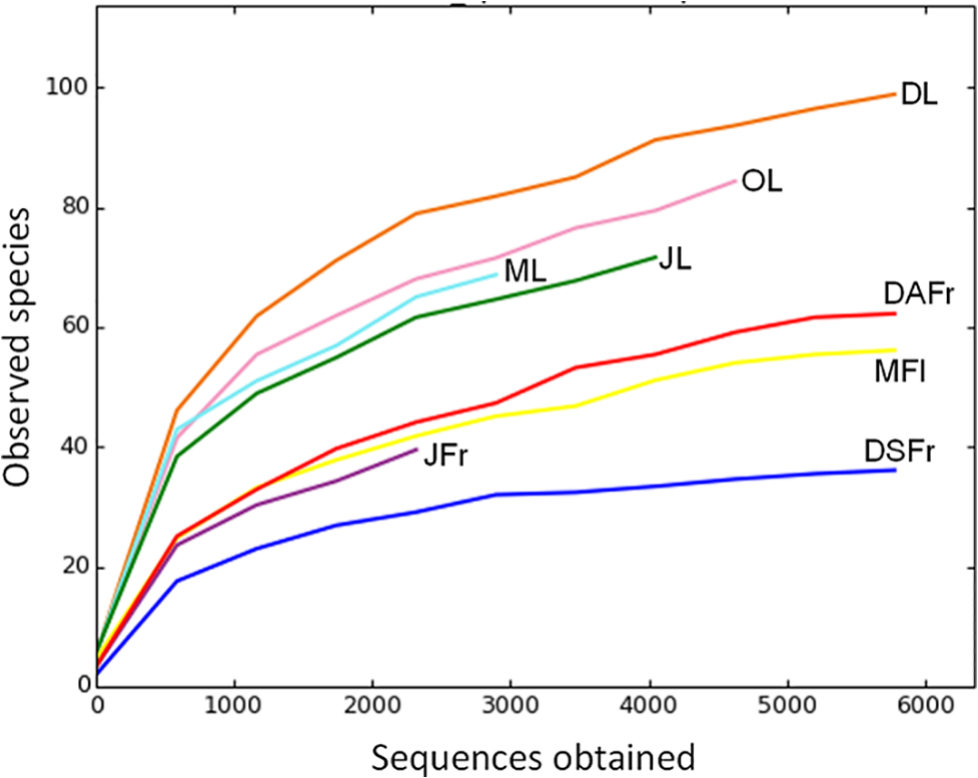
Rarefaction curves at a genetic distance of 3%. Determined for May leaves (ML), June leaves (JL), October leaves (OL), December leaves (DL), May flowers (MFl), June fertilized fruitlets (JFr), December asymptomatic fruits (DAFr) and December symptomatic fruits (DSFr).

**Fig 2:**
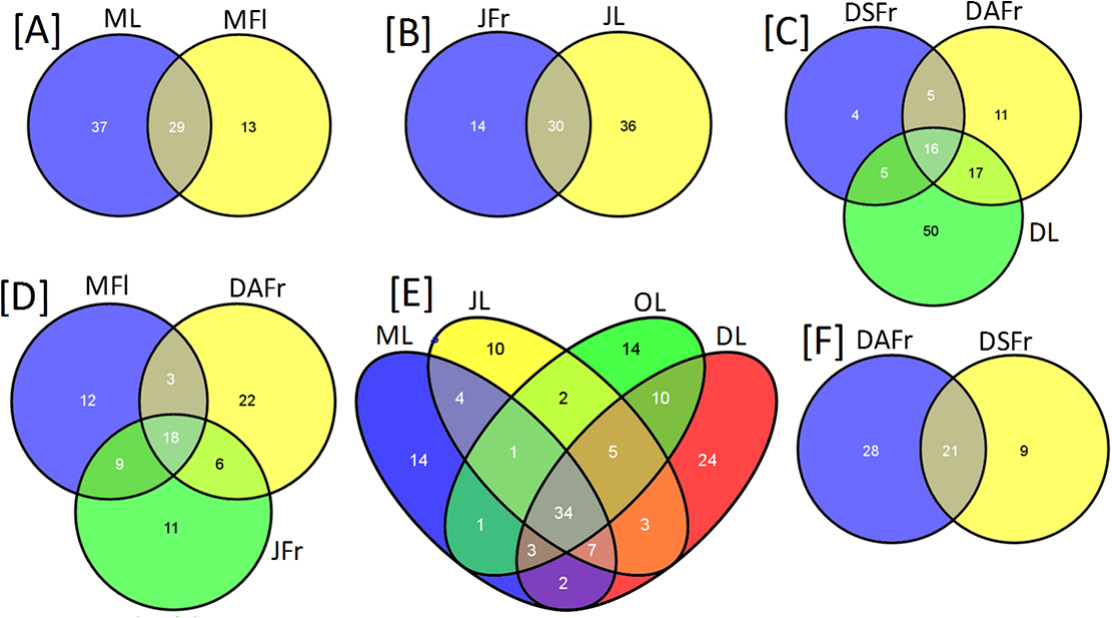
Venn Diagrams reporting the number of OTUs shared among investigated olive sample types in different possible combinations. Analyzed samples included May leaves (ML), June leaves (JL), October leaves (OL), December leaves (DL), May flowers (MFl), June fertilized fruitlets (JFr), December asymptomatic fruits (DAFr) and December symptomatic fruits (DSFr).

The rarefaction analysis assigned to 97% of OTUs similarity showed the achievement of the saturation zone for all samples, suggesting that the great majority of the OTUs was detected in the present study (Fig 1). The number of detected OTUs varied between 37 in symptomatic fruits collected in December (DSFr) and 117 in December leaves (DL). A general higher number of OTUs was detected in leaves compared to May flowers (MFl), JFr and DSFr (Fig 2A, 2B and 2C). Furthermore, leaves were characterized by a higher number of unique OTUs that were not recovered from the other samples of fruits and flowers (Fig 2C). On leaves, the number of OTUs progressively increased from May to December (Fig 2E). With regard to ripe fruits a higher number of OTUs was revealed on DAFr compared to DSFr (Fig 2F).

In agreement with OTU numbers, Shannon’s Diversity Index and the Chao1 estimate based on alpha diversity indexes, achieved their highest values for leaves against MFl, JFr, DSFr and DAFr (Table 1). No big differences were detected for leaves according to the sampling period. As regards flowers evolving into fruits, higher Shannon’s Diversity values were determined for MFl compared to DSFr and DAFr, suggesting a uniform distribution of abundance amongst species. In particular, the lowest value was achieved for DSFr due to the large prevalence of the genus *Colletotrichum* (Table 1).

**Fig 3:**
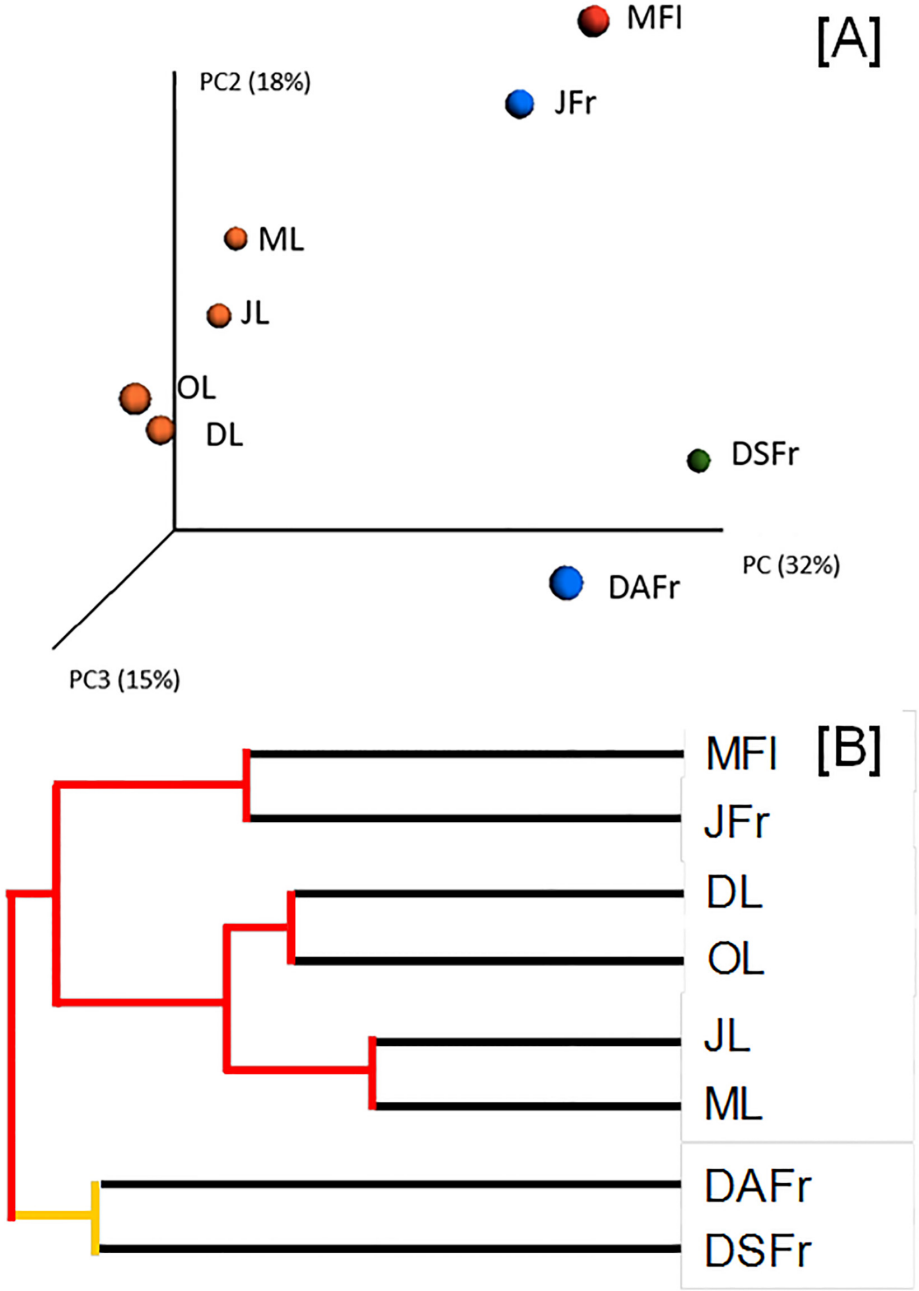
Unweighted UniFrac distance PCoA (A) and Jackknife dendrogram (B) of fungal communities. Associated with May leaves (ML), June leaves (JL), October leaves (OL), December leaves (DL), May flowers (MFl), June fertilized fruitlets (JFr), December asymptomatic fruits (DAFr) and December symptomatic fruits (DSFr). In B red and yellow colors of nodes indicate 75–100% and 50–75% of bootstrap support, respectively.

Beta diversity analysis revealed a clear separation of samples according to sample type. Principal Coordinates Analysis (PCoA) showed a close association between leaf samples that were clearly segregated from all other samples. Interestingly, a close association was revealed between MFl and JFr (Fig 3A). Fungal population on ripe fruits was quite different on DSFr and DAFr but, overall, clearly different compared to other sample types. Results of the PCoA analyses were confirmed by the UPGMA tree (Fig 3B).

### Olive Fungal Community Structure

**Fig 4:**
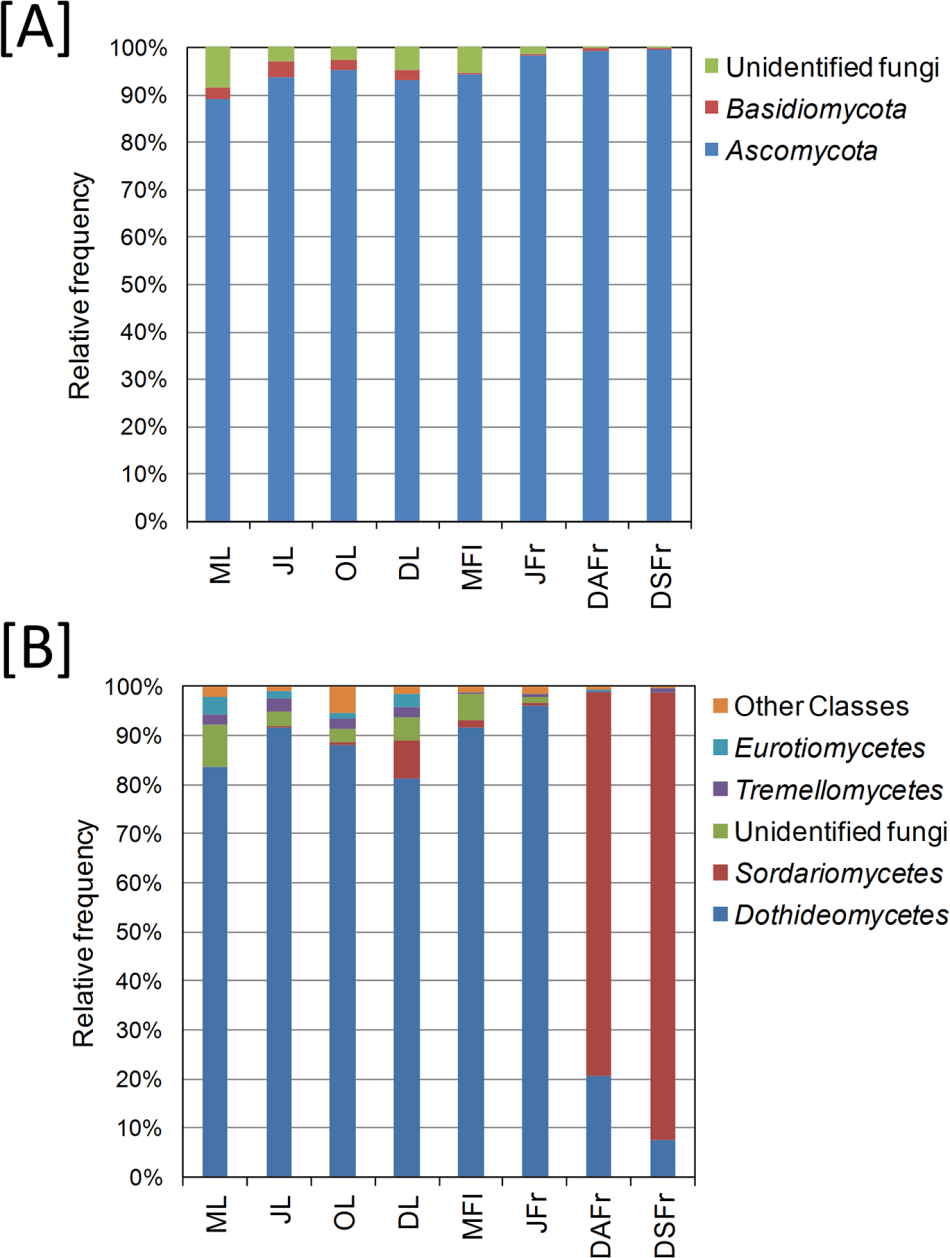
Relative abundance of different fungal phyla (top) and classes (bottom). Detected in May leaves (ML), June leaves (JL), October leaves (OL), December leaves (DL), May flowers (MFl), June fertilized fruitlets (JFr), December asymptomatic fruits (DAFr) and December symptomatic fruits (DSFr).

According to the analysis of the complete ITS2 data set, members of the phylum *Ascomycota* dominated in all samples and accounted for 95.2% of the total number of detected sequences followed by unidentified fungi (3.6%) and *Basidiomycota* (1.5%). In the different samples the incidence of *Ascomycota* varied between 88.6 (May Leaves, ML) and 99.3% (DSFr and DAFr) and was generally higher in fruits compared to flowers and leaves (Fig 4A). *Basidiomycota* accounted for 2.5–3.5% of the reads on leaves but were almost completely absent on flowers and fruits. Similarly, sequences ascribed to non-identified fungi were more abundant on leaves and flowers (4.8–8.7%) and almost completely absent on fruits.

*Ascomycota* reads were largely members of the *Dothideomycetes* and *Sordariomycetes* classes (Fig 4B). The first class predominated on leaves (80.9–91.3%), MFl (91.5%) and JFr (96.4%) while members of the *Sordariomycetes* class were the most abundant on ripe fruits accounting for 76.8 and 91.1% of the reads on DAFr and DSFr, respectively (Fig 4B). *Tremellomycetes* (*Basidiomycota*) and *Eurotiomycetes* (*Ascomycota*) accounted for 0.2–2.7 and 0.1–3.5% of the total reads, respectively. All other detected fungal classes accounted for only 1.7% of the reads (Fig 4B).

**Fig 5:**
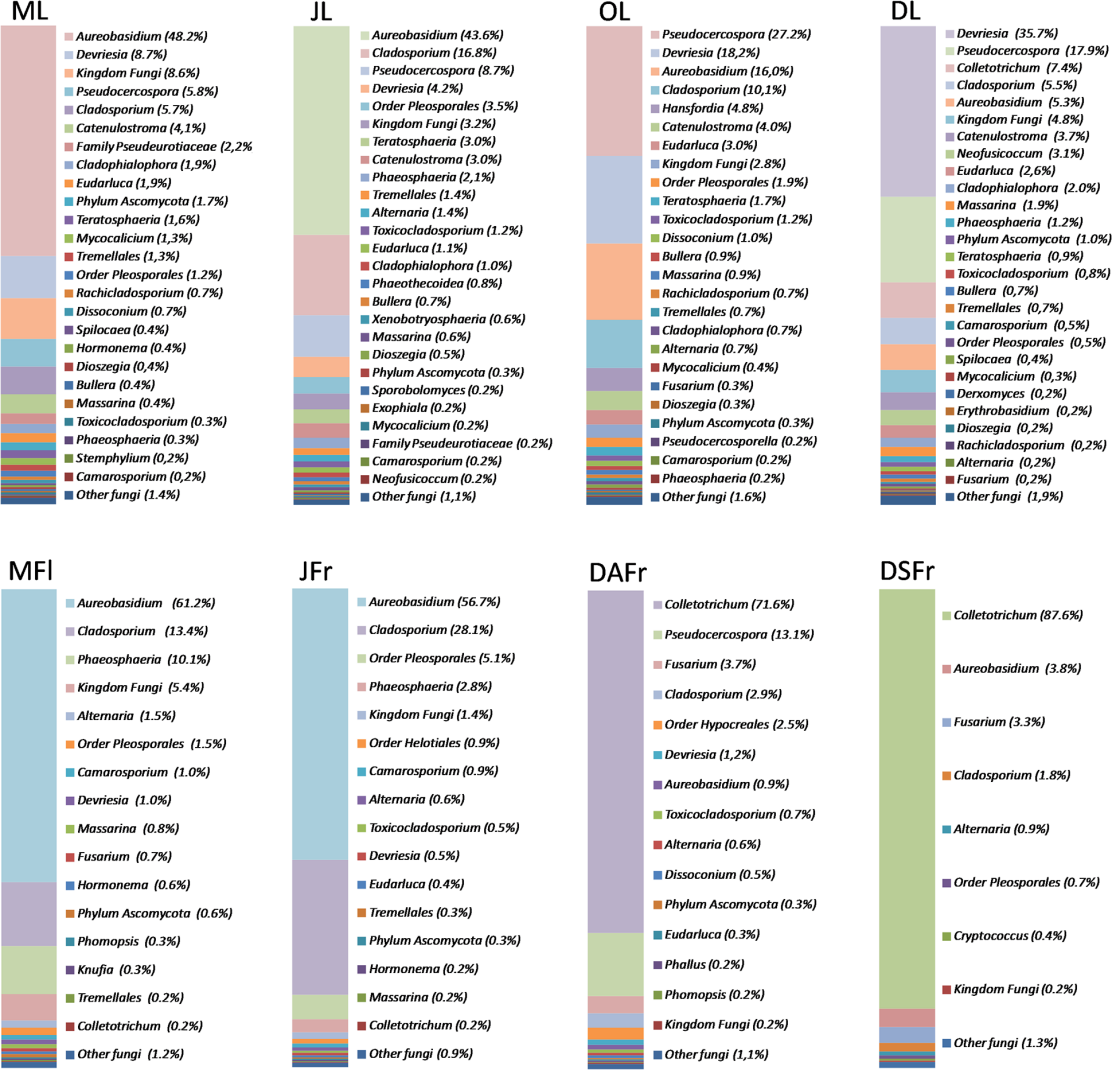
Relative abundance of fungal genera. Detected in May leaves (ML), June leaves (JL), October leaves (OL), December leaves (DL), May flowers (MFl), June fertilized fruitlets (JFr), December asymptomatic fruits (DAFr) and December symptomatic fruits (DSFr). Fungal genera representing less than 0.2% of the total population in each sample, were considered as a single *taxa* and labeled as "Other fungi". In each column fungal genera are listed according to their abundance.

Detected OTUs were associated with a total of 117 different *taxa*. Most of these (103) were identified at the level of genus. However, 1, 2, 2, 4, and 5 *taxa* were only associated with fungi at the level of *Kingdom*, *Phylum*, *Class*, *Order* and *Family*, respectively, due to the lack of closely related sequences in genetic databases. Despite the high number of *taxa* identified, few genera accounted for most reads. Looking at all analyzed samples, the most abundantly detected genus was *Aureobasidium* (31.70%) followed by *Colletotrichum* (19.10%), *Pseudocercospora* (12.70%), *Cladosporium* (12.70%), and *Devriesia* (9.50%) (Fig 5). However, the relative frequency of these genera was not consistent among samples nor during sampling periods. The genus *Aureobasidium* had its highest incidence (43.6–61.2%) in ML, JL and MFl and decreased to 5.3, 3.9 and 0.9% in DL, DSFr and DAFr, respectively (Fig 5). In contrast, *Colletotrichum*, which showed almost no presence until June, started to rise in OL (7.4%) and had its peak in DAFr (70.7%) and DSFr (87.6%). The genus *Pseudocercospora* showed a consistent presence in all leaf samples and its population reached the highest relative frequency in October (30.3%). This genus was also detected on DAFr (14.4%), but not on MFl, JFr and DSFr. The genus *Cladosporium* was detected in all samples but was more abundant in JFr (28.1%), JL (16.8%) and MFl (13.4%). On the contrary, it represented only 1.8 and 2.8% of the reads on DSFr and DAFr, respectively. Finally, the genus *Devriesia*, was almost exclusively detected on leaves and was particularly abundant in December and October, accounting for 35.7% and 18.2% of the total reads, respectively (Fig 5).

### Identification of Fungal Species

**Fig 6:**
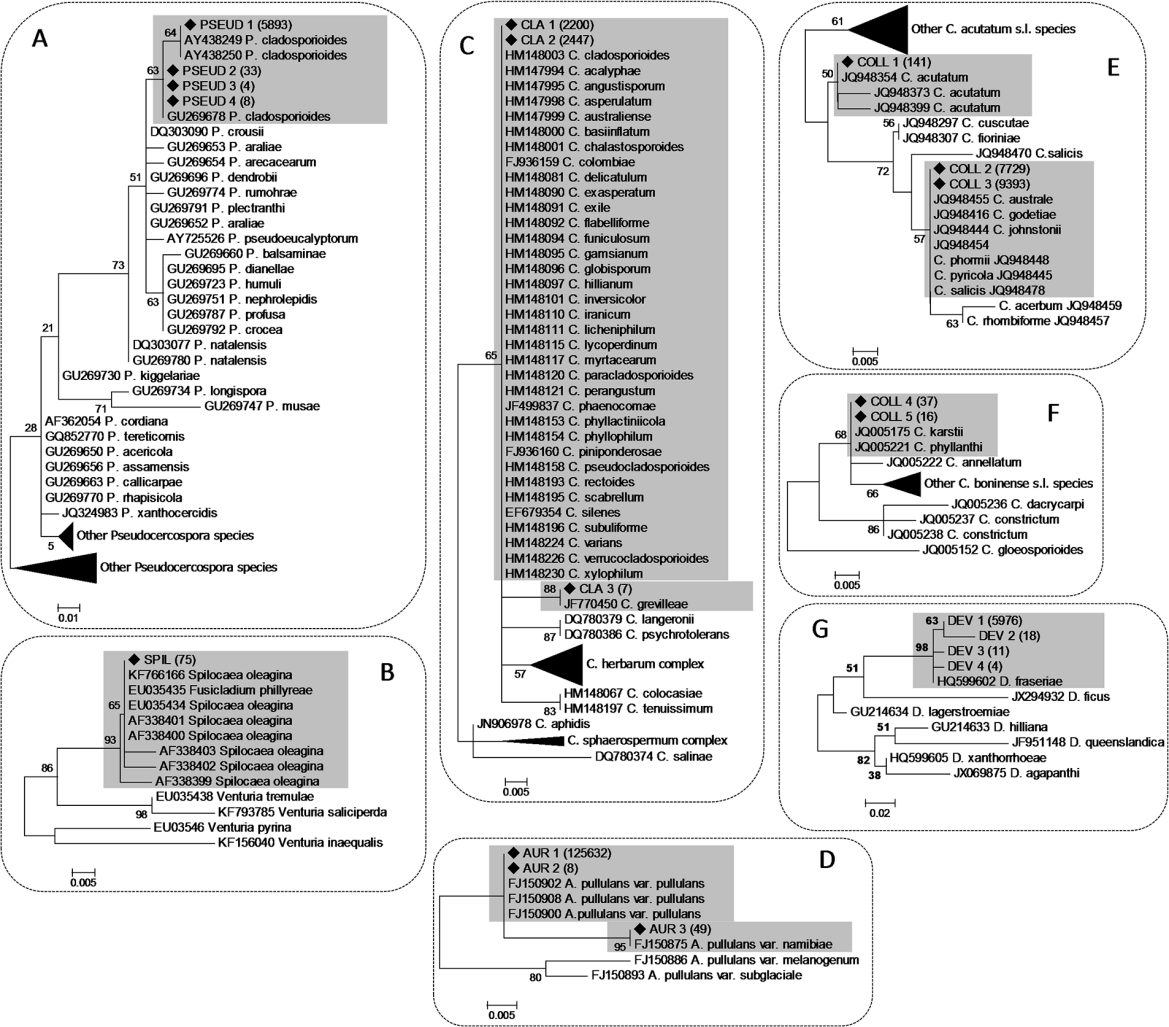
Phylogenetic trees built using unique sequences representative of sequence types (STs) of the most relevant fungal genera in terms of both abundance and/or relevance as olive fungal pathogens, along with validated reference sequences. Reference sequences were from Pseudocercospora spp. (Crous et al. 2013) (A), Cladosporium spp. (Bensch et al., 2013) (C), *Aureobasidium* spp. (Zalar et al., 2008) (D), *Colletotrichum acutatum s*.*l*. (Damm et al., 2012a) (E), *Colletotrichum boninense* s.l. 2012 (Damm et al. 2012b) (F), and *Devriesia* spp. (Li et al. 2013) (B). Sequences of species closely related to *Spilocaea oleagina* were sourced in GenBank (B). Grey highlighted boxes contain STs identified in the present study (♦) and genetically close reference species with which they were associated. Numbers in parentheses along with STs indicate the number of identical sequences, represented by each ST. Numbers on nodes represent the posterior probabilities for the maximum likelihood method.

The manual BLAST analysis of STs confirmed taxonomic assignments of detected OTUs. In addition, phylogenetic analysis of ITS2 sequences of *Aureobasidium* spp., *Colletotrichum* spp., *Cladosporium* spp., *Pseudocercospora* spp., *Devriesia* spp. and *Spilocaea* spp. enabled the identification of most detected STs at the level of species or their association with a restricted number of related species (S1 Table). Three different STs were associated with the genus *Aureobasidium* (Fig 6D). A largely prevalent ST (AUR 1) accounting for 12,562 reads clustered with *A*. *pullulans* var. *pullunans* [39]. The same groups also contained a rare sequence type (AUR 2) differing in a single nucleotide. The other ST (AUR 3) was identified as *A*. *pullulans* var. *namibiae* (Fig 6D).

Five different STs of *Colletotrichum* spp. were detected and found to belong to *C*. *acutatum s*.*l*. (Fig 6E) and *C*. *boninense s*.*l*. (Fig 6F). Among *C*. *acutatum s*.*l*., an ST accounting for 141 reads (COLL 1) was identified as *C*. *acutatum* s.s. while the other two STs (COLL 2 and COLL 3) accounting for 7,929 and 9,393 reads, respectively, clustered with a group of genetically related species which also comprised *C*. *godetiae* (Fig 6E). The two STs clustering within *C*. *boninense s*.*l*. were associated with the species *C*. *karstii* and *C*. *phyllanthi* (Fig 6F).

Similarly, three STs were detected in the genus *Cladosporium* and according to their phylogenetic collocation were found to belong to the *C*. *cladosporioides* complex [40]. For the two largely prevalent sequences (CLA 1 and CLA 2) accounting for 2,200 and 2,4476 reads, respectively, it was not possible to deepen the level of identification due to the high number of species having identical or very similar ITS2 sequences within the complex (Fig 6C). On the contrary, a third rare sequence (CLA 3) accounting for 7 reads was identified as *C*. *grevilleae* (Fig 6C).

Four different STs of *Pseudocercospora* differing for single indels’ were identified as *P*. *cladosporioides* since they were clearly differentiated from all other recognized species of the genus (Fig 6A). A ST (PSEUD 1) was largely prevalent accounting for 5,892 reads while other sequences were rarely detected. Similarly, four different STs were associated with the species *D*. *fraseriae* in light of a 98–99% sequence identity but, a single sequence (DEV 1) accounting for 5976 reads, was by large the most abundant (Fig 6G). Finally, a single ST was detected for the genus *Spilocaea* and was identical to reference sequences of *S*. *oleagina*, a major fungal pathogen of olive, and *Fusicladium phillyreae* (Fig 6B).

## Discussion

In the present study, the fungal diversity associated with olive leaves, flowers and fruits was investigated at different phenological stages using a metabarcoding approach. Analyses revealed a rich fungal community with 195 different OTUs which were identified up to genus level in most of the cases. Furthermore, phylogenetic analyses permitted the identification of many STs at the level of species. A conspicuous number of OTUs, however, was only identified at the level of *Phylum*, *Order*, *Class* or *Family* suggesting the existence of several rare or still unknown fungal *taxa*. At least a part of these putative species is likely to represent uncultivable *taxa* and their ecological role is currently unknown. These findings highlight the importance of the metabarcoding approaches in determining the complex microbial populations associated with plant surfaces since only a limited part of the available genetic variation can be investigated using traditional culturing methods.

Regardless of the sample type or period, *Ascomycota* was clearly the dominant phyla (93.6%) followed by unidentified fungi and *Basidiomycota*. Within *Ascomycota*, the class *Dothideomycetes* (70.1%) was the most abundant followed by *Sordariomycetes* (22.4%). The high incidence of the former class was primarily determined by the abundant detection of the genera *Aureobasidium* (Order *Dothideales*) and *Cladosporium*, *Pseudocercospora* and *Devriesia* (Order *Capnodiales*), while the class *Sordariomycetes* was almost completely represented by fungi of the genus *Colletotrichum* which were abundantly detected in both DAFr and DSFr. In agreement with our study, this fungal class was also predominant on grape leaves and grape must [18]. Indeed *Dothideomycetes* is one of the largest and most significant classes within *Ascomycota*, which also comprises thousands of plant pathogen species [41].

A higher level of diversity was revealed for leaves in comparison with flowers and fruits, regardless of the sampling period. In agreement with our results, fungal and bacterial populations on flowers and fruits were at least ten times less than those on leaves in *Sesamum* and *Gossypium* plants [42]. This result may be related to the fact that leaves have a higher surface/volume ratio compared to fruits, cover most of the plant surface, are always present on the plant (olive is an evergreen tree), and each single leaf has a life cycle of approximately 18 months against the 5–6 months of the flowers evolving in fruits. Since leaves are always present on the tree, they can establish a sort of balanced community. In addition, leaves represent a different ecological niche in terms of surface composition and landscape [43, 44]. Unlike leaves, the fungal community changed significantly from fruitlets to mature fruits and this was also associated with a sharp decline in fungal biodiversity (richness and evenness) mainly because of the epidemic outbreak of *Colletotrichum* spp. in mature symptomatic and asymptomatic fruits. In general, the olive fungal community on fruitlets was similar to that of the organs from which they originated (flowers) and progressively evolved in the fruit community. In agreement with our data, different anatomical parts of tomato were characterized by distinct microbial communities, but flowers and fruit shared a few bacterial *taxa* that were not detected in other parts of the plant [19]. It has been suggested that ovaries developing into the flesh of fruits represent a desired habitat for microbial colonization, potentially providing both long-term resources and shelter for the microbes able to enter [45]. To some extent, a similar phenomenon is known to occur in humans since a new-born’s gut microbial community is initially similar to that of the mother and then progressively evolves [46].

In the present study, amplicons were analyzed with QIIME using a high quality filtering set up in order to minimize the impact of sequencing errors and achieve a reliable identification of OTUs. Indeed, the manual BLAST analysis of all detected STs confirmed the reliable identification of OTUs up to the level of genus. Conversely, standard QIIME analyses largely failed in the identification of fungal species. This result was partially expected considering that genetic variation within ITS regions may be very limited or inexistent among closely related species. Consequently, the level of homology commonly suggested in literature and utilized in the present study for picking OTUs (97%) did not enable an accurate identification of species. Furthermore, it is worth mentioning that unreliable annotations of sequences in public DNA repositories remain a serious obstacle to all sequence-based species identification [47]. It should also be considered that a significant part of deposited ITS sequences are not updated and may not reflect recent advancements in fungal taxonomy [48, 49]. However, despite the above limits and biases, the ITS regions are widely accepted as the official fungal DNA barcode marker because they can be easily amplified and sequenced using Sanger and second generation sequencing approaches [5].

To exploit all available genetic variations within the ITS2 region and enable the identification of detected *taxa* with the highest possible level of accuracy, STs associated with the most relevant fungal genera in terms of both abundance and/or relevance as olive fungal pathogens, were subjected to specific phylogenetic analyses along with selected validated reference sequences. This approach proved to be reliable considering that the majority of the STs were identified at the level of species and that the remaining ones were associated with a restricted number of *taxa*. This achievement is very important for the analysis of plant-associated fungi with particular emphasis to pathogens, since related species with very similar ITS sequences can be characterized by completely different behaviors. Consequently, the determination of fungal diversity up to the level of species is essential to study the ecology and biology of fungal pathogens on their own hosts and in relation to other microorganisms. These data may be useful from a practical point of view since they may be used to evaluate the impact of control strategies on pathogens and non-pathogens in the aerial plant surface with the aim of developing new more effective and less impacting means for disease management.

The reliability and accuracy of any phylogenetic approach used to identify fungal species are largely influenced by the comprehensiveness of data on the phylogenetic collocation of analyzed *taxa* and by the consequent availability of validated reference sequences. An accurately validated database has been recently released but it still does not cover all currently known genetic variations within the fungal kingdom [48]. However, several comprehensive studies have recently clarified the taxonomy in important fungal groups and many others are likely to come out in the near future. It is also possible to anticipate the future use of more variable markers as barcode genes in metabarcoding analyses to enable a higher level of discrimination among species [32]. However, the single copy nature of currently available marker genes is likely to provide lower levels of sensitivity compared to the multi-copy ITS regions. Furthermore, difficulties in designing reliable universal primers and the lower number of available reference sequences in genetic databases may represent an issue in species identification [5].

A conspicuous part of sequences detected in the present study was associated with well known olive fungal pathogens. The abundance detection of the genus *Colletotrichum* was expected considering that trials were conducted on the Gioia Tauro plain (southern Italy) which is characterized by recurrent anthracnose outbreaks. The detection of this genus in May, June and October with a low level of population suggests that flowers, leaves and unripe fruits may harbour latent infections. It has been suggested that latent infections in developing fruits during spring may favor the survival of the pathogen later in the hot and dry summer, but the relevance of these infections to *Colletotrichum* epidemic outbreaks is not yet well defined [21, 50, 51]. On the other hand, the high incidence of *Colletotrichum* species in DL represents a confirmation of reported data on the importance of fruits in the promotion of leaf infections [50, 52]. Among sequences associated with the species complex of *C*. *acutatum s*.*l*., a ST was identified as *C*. *acutatum* s.s. while other two STs accounting for most of the detected reads clustered with a group of genetically related species which also comprised *C*. *godetiae*. Since this latter species is known as the causal agent of olive anthracnose in southern Italy it is likely that detected sequences belonged to it although the ITS2 region does not enable its differentiation from closely related species [33, 53]. Similarly, STs clustering within the species complex of *C*. *boninense s*.*l*. are likely to belong to *C*. *karstii* which has been recently reported on olives in southern Italy [21]. The detection of *C*. *acutatum s*.*s*. on olives in southern Italy confirms results of a recent metabarcoding investigation with *Colletotrichum* specific primers [21]. Interestingly, *C*. *acutatum s*.*s*. had been not associated with olive anthracnose in Italy until recently, suggesting an ongoing process of evolution in the populations of this important plant pathogen [53]. The abundant detection of *Colletotrichum* spp. on ripe olives was expected for symptomatic fruits but it was to some extent surprising for the asymptomatic ones (71.6% of the total reads). This result may suggest a conspicuous colonization of olive tissues before the appearance of symptoms and the consequent competitive exclusion of other fungi.

Another widely detected plant pathogen was identified as *P*. *cladosporioides* i.e. the causal agent of olive cercosporiosis. The high incidence of *P*. *cladosporioides* on leaves in the investigated environment seems to be remarkable and may suggest a primary role of this pathogen in the defoliation of trees. By contrast and quite surprisingly the incidence of *S*. *oleagina*, commonly considered as the main causal agent of olive defoliation, was low [54]. A possible explanation is the poor saprophytic and epiphytic ability of this fungal species; moreover, it is reported that the cultivar Ottobratica is highly resistant to the peacock spot disease. Infections by *P*. *cladosporioides* are generally considered to take place later in comparison with those caused by *S*. *oleagina* and therefore they should be limited to old, mature leaves of lower branches. However, in agreement with our data, field surveys conducted in southern Italy showed that the fungus can be active throughout the year [54, 55]. The high incidence on DAFr (14.4% of the total reads) also suggests that infections on these organs may be quite common, although *P*. *cladosporioides* is mainly considered a leaf pathogen. In this regard, the sampling area (Gioia Tauro plain, southern Italy) is characterized by humid autumn conditions and it is reported that the severity of fruit infection is related to persistent humid and mild weather during the last three months before harvest [56].

At lower abundance, other olive pathogens represented by *Fusarium* spp. were primarily detected in asymptomatic and symptomatic ripe fruits. Although the analysis of sequences did not enable the identification of the species due to the complexity of the genus [57], it is reported that *Fusarium* species can be responsible for olive rots [3]. Interestingly, *Fusarium* spp. were also detected with a lower incidence on flowers suggesting that the fungus may colonize and establish latent infection on these organs. Other detected fungi that are likely to act as plant pathogens were associated with the genera *Neofusicoccum* and *Alternaria*. Both genera comprise species that can cause leaf and fruit infections on olive [3]. In particular, according to BLAST analyses, two species of *Neofusicoccum*, *N*. *parvum* and *N*. *mediterraneum*, were detected on olive samples with the first accounting for a higher incidence. Additionally, *Stemphylium* spp. and *Phomopsis* spp., two genera comprising species that may be regarded as causal agents of minor olive diseases [3] were detected in the current work with a very low incidence. Finally, some of the detected OTUs were associated with fungal genera (*Selenophoma* spp., *Teratosphaeria* spp. and *Cladophialophora* spp.) comprising relevant pathogens on other plant species, but their role on olive is completely unknown.

The genus *Aureobasidium* spp. was one of the most abundant on leaves, MFl and JFr and to a lesser extent was also detected in DAFr and DSFr. Detected STs were largely associated with *A*. *pullulans* var. *pullulans* and to a lesser extent with *A*. *pullulans* var. *namibiae*. The abundant presence of *A*. *pullulans* var. *pullulans* in the olive canopy was expected while, to the best of our knowledge, *A*. *pullulans* var. *namibiae* has never been reported as a colonizer of plant tissues [39]. *Aureobasidium pullulans* is a ubiquitous yeast-like fungus that can colonize almost all environmental niches including soil, water, air, and limestone. It has been reported as one on the most abundant fungal colonizers of phyllosphere and carposphere in a number of different plant species and may be present as both epiphyte and endophyte [58, 59]. However, apart from a few reports in which it has been demonstrated to act as a pathogen on overripe fruits [60, 61], it is generally considered a non-pathogen and has been widely exploited as a bio-control agent [62, 63]. On olives, it was mainly reported as a component of a complex of fungi causing sooty mold i.e. an epiphytic growth of different saprophytic fungi forming a black film which covers leaves, fruits, twigs and branches [3].

Another widely detected genus in olive samples was *Cladosporium* spp. with three different STs. Two STs were largely prevalent and were associated with the *C*. *cladosporioides* species complex since available genetic variation within the ITS2 region does not enable the differentiation of most species within this complex [64]. A third ST accounting for few sequences was associated with the species *C*. *grevilleae*. The species complex of *C*. *cladosporioides* comprises very common cosmopolitan saprophytic fungi. *C*. *cladosporioides* often occurs as a secondary invader of necrotic tissues or as a weak pathogens in many different host plants [65]. However, some strains of *C*. *cladosporioides* have been also reported as effective bio-control agents [66]. Together with *A*. *pullulans*. *C*. *cladosporioides* had been classified among sooty mold fungi that grow on the surface of leaves and other plant organs covered with insect or physiological honeydew in different plant species including olive [3, 65, 67]. In favorable conditions, both fungal species may produce a compact sooty thallus on the fruit surface even in the absence of honeydew [68].

According to our data, the genus *Devriesia* is one of the most abundant fungal colonizers of olive canopy although it was almost exclusively detected on leaves and was particularly abundant in December and October. The Mediterranean climate characterized by hot and dry summers followed by mild temperatures and high rainfall incidence in autumn and winter, may have plaid a role in this temporal distribution. Interestingly, the genus *Devriesia* has never been reported as an olive fungal colonizer. It is possible that it had been formerly identified as a *Cladosporium* species since the genus *Devriesia* also contains species previously ascribed to this genus [69]. Sequences detected in the present study were associated with *D*. *fraseriae* a species described in 2010 to accommodate a fungus isolated from leaves of *Melaleuca* sp. in Australia. Considering its high incidence on olive leaves, it may be assumed to have a role in sooty mold along with other species of the *Capnodiaceae* family [70].

The high incidence of *Aureobasidium* spp., *Cladosposorium* spp. and *Devriesia* spp. in olive phyllosphere and to a lesser extent in the olive carposphere suggests a primary role in the structure of fungal communities associated with olive trees and a possible competitive action against fungal plant pathogens. Furthermore, other genera detected with a low frequency (*Hormonema* spp., *Dissoconium* spp., *Hansfordia* spp., *Bullera* spp. and *Dioszegia* spp.) may have competitive actions since they comprise species with well documented antagonistic functions.

In conclusion results of the present study provide a comprehensive picture of the fungal diversity in the olive phyllosphere and carposphere. The majority of the detected sequences were identified up to the level of species making it possible to support assumptions regarding their role on the olive aerial plant surface. However, many others were not associated with specific *taxa* and, even if identified, their significance remains to be interpreted. Altogether, this study reinforces the importance of investigating fungal biodiversity in olive culture and highlighting the need for more detailed analyses in this field. The olive fungal consortia showed to contain both beneficial and phytopathogenic microorganisms that can have a significant impact on olive productions. Beneficial colonizers, either epiphytes or entophytes, can be further explored as antagonists of the important pathogens of olive, and possibly developed as effective biocontrol agents.

## Supporting Information

S1 Table Sequence types (STs) and corresponding associated fungal *taxa* representative of the most abundant genera in the olive canopy. (DOCX)
