# Supplementary material for: Metabarcoding Analysis of Fungal Diversity in the Phyllosphere and Carposphere of Olive (Olea europaea)
Source: PLoS One. 2015 Jul 1;10(7):e0131069. doi: 10.1371/journal.pone.0131069 (PMC4489200; doi:10.1371/journal.pone.0131069)
Supplement: S1 Table — (DOCX) [file pone.0131069.s001.docx]

**S1 Table**. Sequence types (STs) and corresponding associated fungal *taxa* representative of the most abundant genera in the olive canopy

| **Sequence types (ST)** | **ITS2 sequences** | **Associated taxa** |
| --- | --- | --- |
| AUR 1 | CACCACTCAAGCTATGCTTGGTATTGGGCGTCGTCCTTAGTTGGGCGCGCCTTAAAGACCTCGGCGAGGCCACTCCGGCTTTAGGCGTAGTAGAATTTATTCGAACGTCTGTCAAAGGAGAGGAACTCTGCCGACTGAAACC | *Aureobasidium pullulans var. pullulans* |
| AUR 2 | CACCACTCAAGCTATGCTTGGTATTGGGTGCCGTCCTTAGTTGGGCGCGCCTTAAAGACCTCGGCGAGGCCACTCCGGCTTTAGGCGTAGTAGAATTTATTCGAACGTCTGTCAAAGGAGAGGAACTCTGCCGATTGAAACC | *Aureobasidium pullulans var. pullulans* |
| AUR 3 | CACCACTCAAGCTATGCTTGGTATTGGGCGTCGTCCTTAGTTGGGCGCGCCTTAAAGACCTCGGCGAGGCCACTCCGGCTTTAGGCGTAGTAGAATTTATTCGAACGTCTGTCAAAGGAGAGGAAACTCTGCCGACTGAAACC | *Aureobasidium pullulans var. namibiae* |
| CLA 1 | CACCACTCAAGCCTCGCTTGGTATTGGGCAACGCGGTCCGCCGCGTGCCTCAAATCGACCGGCTGGGTCTTCTGTCCCCTAAGCGTTGTGGAAACTATTCGCTAAAGGGTGTTCGGGAGGCTACGCCGTAAAACAACCCCATTTCTAA | *Cladosporium cladosporioides species complex* |
| CLA 2 | CACCACTCAAGCCTCGCTTGGTATTGGGCAACGCGGTCCGCCGCGTGCCTCAAATCGACCGGCTGGGTCTTCTGTCCCCTAAGCGTTGTGGAAACTATTCGCTAAAGGGTGTTCGGGAGGCTACGCGTAAAACAACCCCATTTCTAA | *Cladosporium cladosporioides species complex* |
| CLA 3 | CACCACTCAAGCCTCGCTTGGTATTGGGCAATGCGGTCCGCCGCATGCCTCAAATCGACCGGCTGGGTCTTCTGTCCCCTAAGCGTTGTGGAAACTATTCGCTAAAGGGTGTTCGGGAGGCTACGCCGTAAAACAACCCCATTTCTAA | *C. grevilleae* |
| COLL 1 | CAACCCTCAAGCACCGCTTGGTTTTGGGGCCCCACGGCAGACGTGGGCCCTTAAAGGTAGTGGCGGACCCTCCCGGAGCCTCCTTTGCGTAGTAACTAACGTCTCGCACTGGGATCCGGAGGGACTCTTGCCGTAAAACCCCCAAATTCTTTACA | *Colletotrichum acutatum s.s.* |
| COLL 2 | CAACCCTCAAGCACCGCTTGGTTTTGGGGCCCCACGGCCGACGTGGGCCCTTAAAGGTAGTGGCGGACCCTCCCGGAGCCTCCTTTGCGTAGTAACTAACGTCTCGCACTGGGATCCGGAGGGACTCTTGCCGTTAAACCCCCAAATTTTTCA | *Colletotrichum godetiae* |
| COLL 3 | CAACCCTCAAGCACCGCTTGGTTTTGGGGCCCCACGGCCGACGTGGGCCCTTAAAGGTAGTGGCGGACCCTCCCGGAGCCTCCTTTGCGTAGTAACTAACGTCTCGCACTGGGATCCGGAGGGACTCTTGCCGTTAAACCCCCAAATTTTTTCA | *Colletotrichum godetiae* |
| COLL 4 | CAACCCTCAAGCTCTGCTTGGTGTTGGGGCTCTACGGTCGACGTAGGCCCTCAAAGGTAGTGGCGGACCCTCCCGGAGCCTCCTTTGCGTAGTAACATTTCGTCTCGCACTGGGATCCGGAGGGACTCTTGCCGTAAAACCCCCCAATTTTCCAA | *Colletotrichum karstii* |
| COLL 5 | CAACCCTCAAGCTCTGCTTGGTGTTGGGGCTCTACGGTCGACGTAGGCCCTCAAAGGTAGTGGCGGACCCTCCCGGAGCCTCCTTTGCGTAGTAACATTTCGTCTCGCACTGGGATCCGGAGGGACTCTTGCCGTAAAACCCCCCATTTTCCAA | *Colletotrichum karstii* |
| DEV 1 | CACCAATCACGCCTGGCGTGGTATTGGGCGACGCGGCCGTCACACGCCGCGCGCCCCAATGACTCCGGCGGGACGGACCGAATCTCAGCGTTGTGGTTAAAGCCGCTGGCGAGACGGGACGCCCGTGCCGTTAAACAACCCCATTACA | *Devriesia fraseriae* |
| DEV 3 | CACCAATCACGCCTGGCGTGGTGTTGGGCGACGCGGCCGTCACACGCCGCGCGCCCCAATGACTCCGGCGGGACGGACCGAATCTCAGCGTTGTGGTTAAGGCCGCTGGCGAGACGGGACGCCCGTGCCGTTAAACAACCCCCATCACA | *Devriesia fraseriae* |
| DEV 4 | CACCAATCACGCCTGGCGTGGTATTGGGCGACGCGGCCGTGACACGCCGCGCGCCCCAATGACTCCGGCGGGACGGACCGAATCTCAGCGTTGTGGTTAAAGCCGCTGGCGAGACGGGACGCCCCGTGCCGTTAAACAACCCCATCACA | *Devriesia fraseriae* |
| DEV 2 | CACCAATCACGCCTGGCGTGGTATTGGGCCCGACGCGGCCGTCACACGCCGCGCGCCCCAATGACTCCGGCGGGACGGACCGAATCTCAGCGTTGTGGTTAAAGCCGCTGGCGAGACGGGACGCCCGTGCCGTTAAAACAACCCCATTGCA | *Devriesia fraseriae* |
| PSEUD1 | CACCACTCAAGCCTGGCTTGGTATTGGGCGTCGCGGCTCCGCGCGCCTCAAAGTCTCCGGCTGAGCCATTCGTCTCTAAGCGTTGTGGATTTTCTAATTCGCTTCGGGGTGCGGGTGGCCGCGGCCGTTAAATCTTTATTCAAA | *Pseudocercospora cladosporioides* |
| PSEUD2 | CACCACTCAAGCCTGGCTTGGTATTGGGCGTCGCGGCTCCGCGCGCCTTAAAGTCTCCGGCTGAGCCATTCGTCTCTAAGCGTTGTGGATTTTCTAATTCGCTTCGGGGTGCGGGTGGCCGCGGCCGTTAAATCTTTATTCAAA | *Pseudocercospora cladosporioides* |
| PSEUD4 | CACCACTCAAGCCTGGCTTGGTATTGGGCGTCGCGGCTCCGCGCGCCTTAAAGTCTCCGGCTGAGCCATTCGTCTCTAAGCGTTGTGGATTTTCTAATTCGCTTCGGGTGCGGGTGGCCGCGGCCGTTAAATCTTTATTCAAA | *Pseudocercospora cladosporioides* |
| SPIL | TACCCTGGAGCCCTGCTCTGTGATGGGCCCCGTCCTCGCGGACGGGCCCGAAACCCGTGGGCGCCGTCGTCCGGCCCCGAGCGTAGCAAGAGAAATCCCTCGCTCGGAGCGCCTGGCGGCCGGCCGCCCCGAAACCTACTTCTACAA | *Spilocaea oleagina* |
